# Supplementary material for: Anti-correlated feature selection prevents false discovery of subpopulations in scRNAseq
Source: Nat Commun. 2024 Jan 24;15:699. doi: 10.1038/s41467-023-43406-9 (PMC10808220; doi:10.1038/s41467-023-43406-9)
Supplement: Supplementary file 1 — Supplementary Information [file 41467_2023_43406_MOESM1_ESM.pdf]

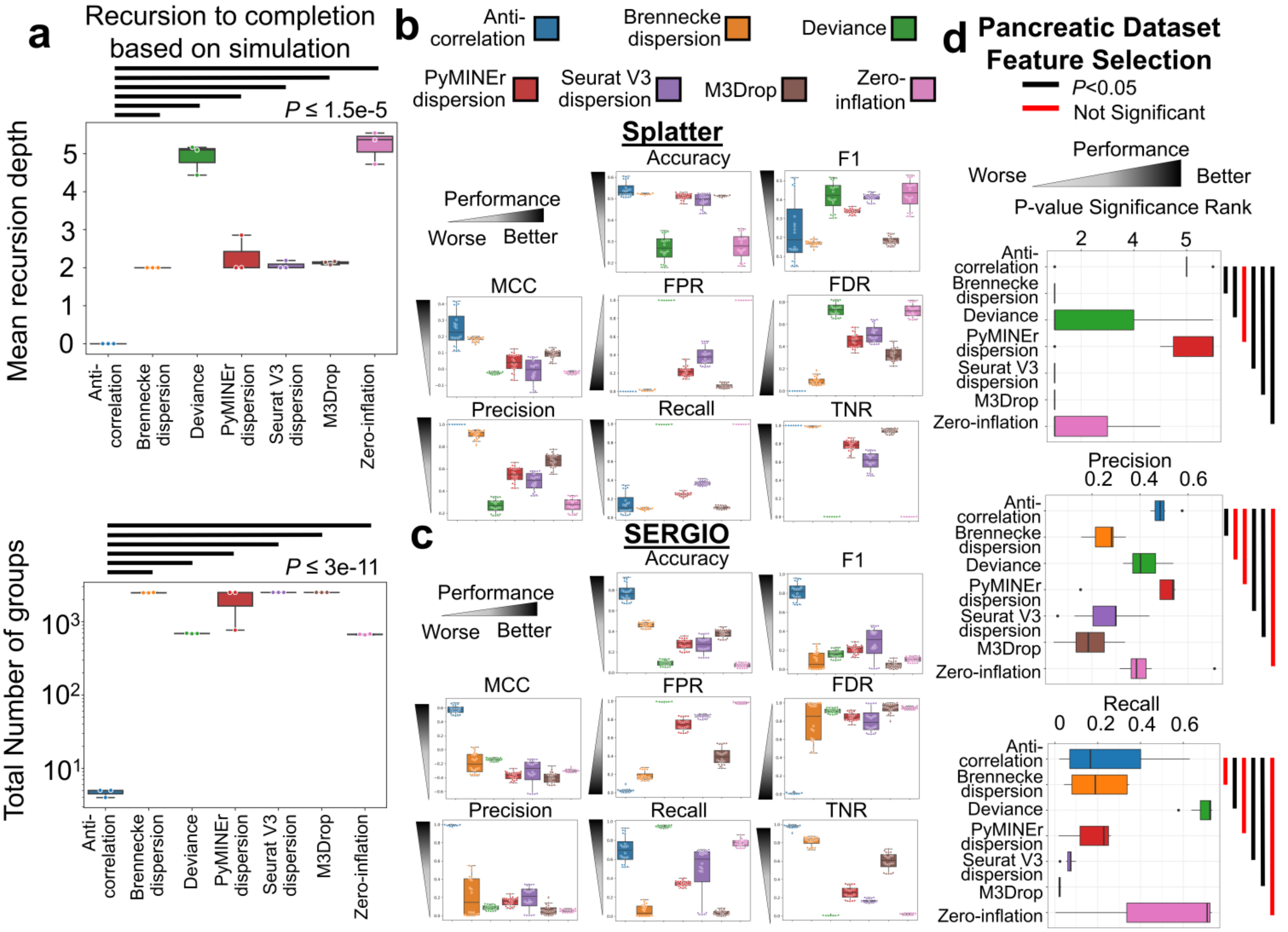

Supplementary Figure 1: Anti-correlation-based feature selection outperforms other methods in recursion-to-completion and feature selection efficacy.

a, Using Splatter simulation of four clusters, all algorithms were allowed to select features and perform locally weighted Louvain modularity-based clustering recursively. Shown are boxplots indicating the mean recursive depth and total number of clusters on a log scale. The anti-correlation algorithm did not allow for any recursive clustering, resulting in fewer clusters identified (*:*P*≤6.2e-6; ***:*P*≤1.7e-9; all ANOVA/TukeyHSD post-hoc comparisons against anti-correlation, n=3 datasets). **b-c**, Taken as a classification problem in which a feature selection algorithm's task is to select detectably differentially expressed genes across clusters, we quantified each algorithm's accuracy, F1 score, Mathew's Correlation Coefficient (MCC), false positive rate (FPR), false discovery rate (FDR), precision, true negative rate (TNR), and recall (n=20 datasets each). **b**, Boxplots of classification metrics (panels) by feature selection approach (colored boxes) using Splatter simulations[16]. **c**, Boxplots indicating the performance of each features selection method (colored boxes) for each metric (panels), using SERGIO, gene regulatory network based simulations[18]. All boxplots show lines that extend to minimum and maximum, with the box bounds from 25th-75th percentile, and center denoting the median. **d**, Using 7 pancreatic datasets[3, 26-30], each algorithm's selected features was analyzed for significance with pancreatic tissue enrichment via gProfiler and the human protein atlas[31, 32]; displayed are boxplots of the "best" pancreatic pathway by p-value comparing this pathway's rank p-value, precision, and recall (n=7 datasets). Exact p-values available in source data. Source data are provided as a Source Data file.

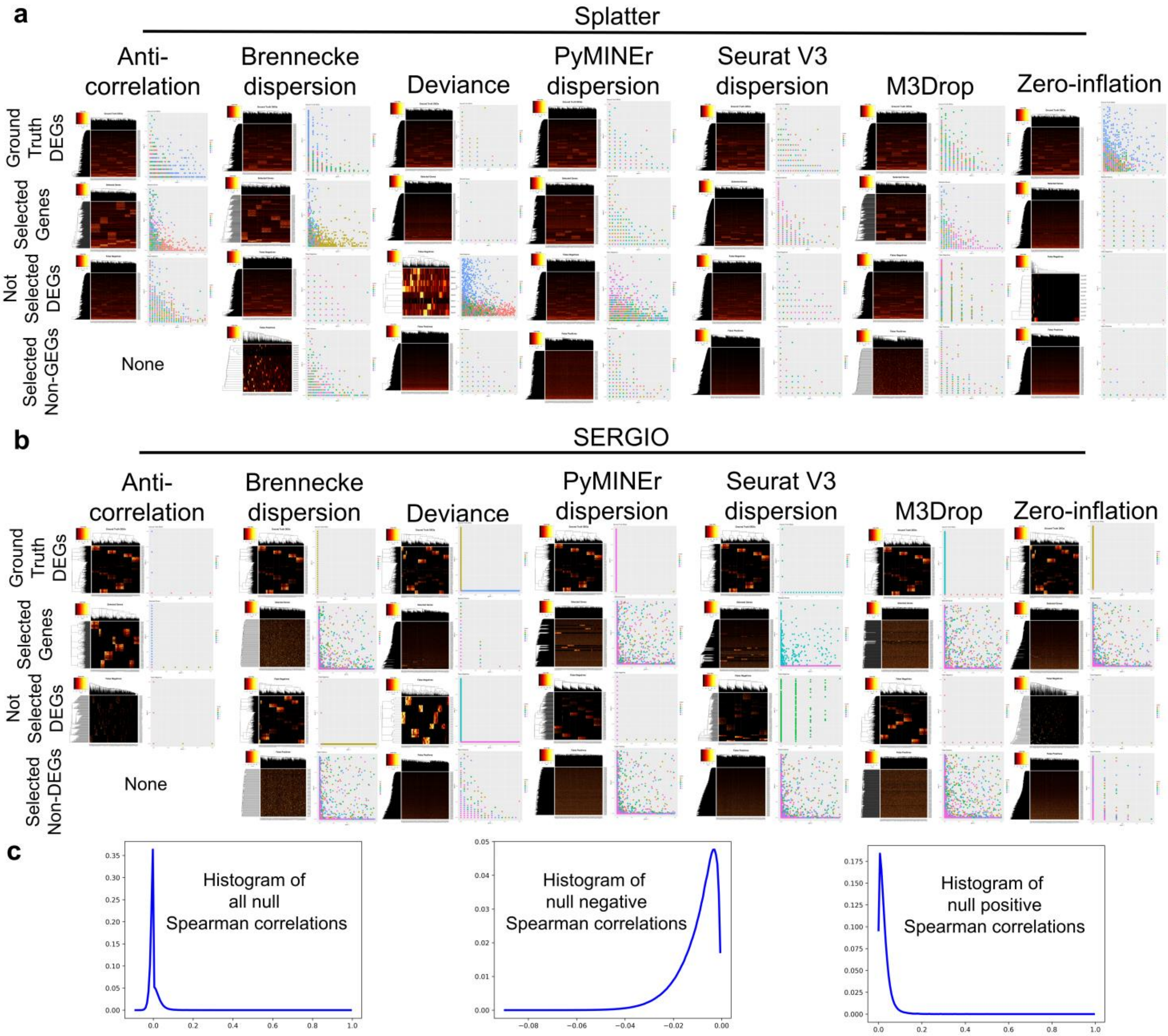

Supplementary Figure 2: Examples of Splatter and SERGIO simulations, and feature selection.

a,b, For both simulation paradigms (**a**) Splatter and (**b**) SERGIO, heatmaps are shown for the ground truth differentially expressed genes (DEGs), the selected-genes, non-selected DEGs, and selected genes that are not differentially expressed. Next to the heatmaps are gene-gene scatter plots of randomly selected genes from the indicated class (row) for the feature selection algorithms (columns). Points indicate an individual cell's expression of random gene-x and gene-y for the designated gene class and algorithm, color-coded by the simulated cluster. (**a**) Splatter DEGs show widespread co-expression of DEGs within all clusters, while (**b**) SERGIO allows for cluster specific expression of DEGs. (**c**) An example histogram of null distribution patterns of Spearman rhos on shuffled datasets shows that, even on shuffled data with no true positives, negative rhos follow a different distribution than positive rho values.

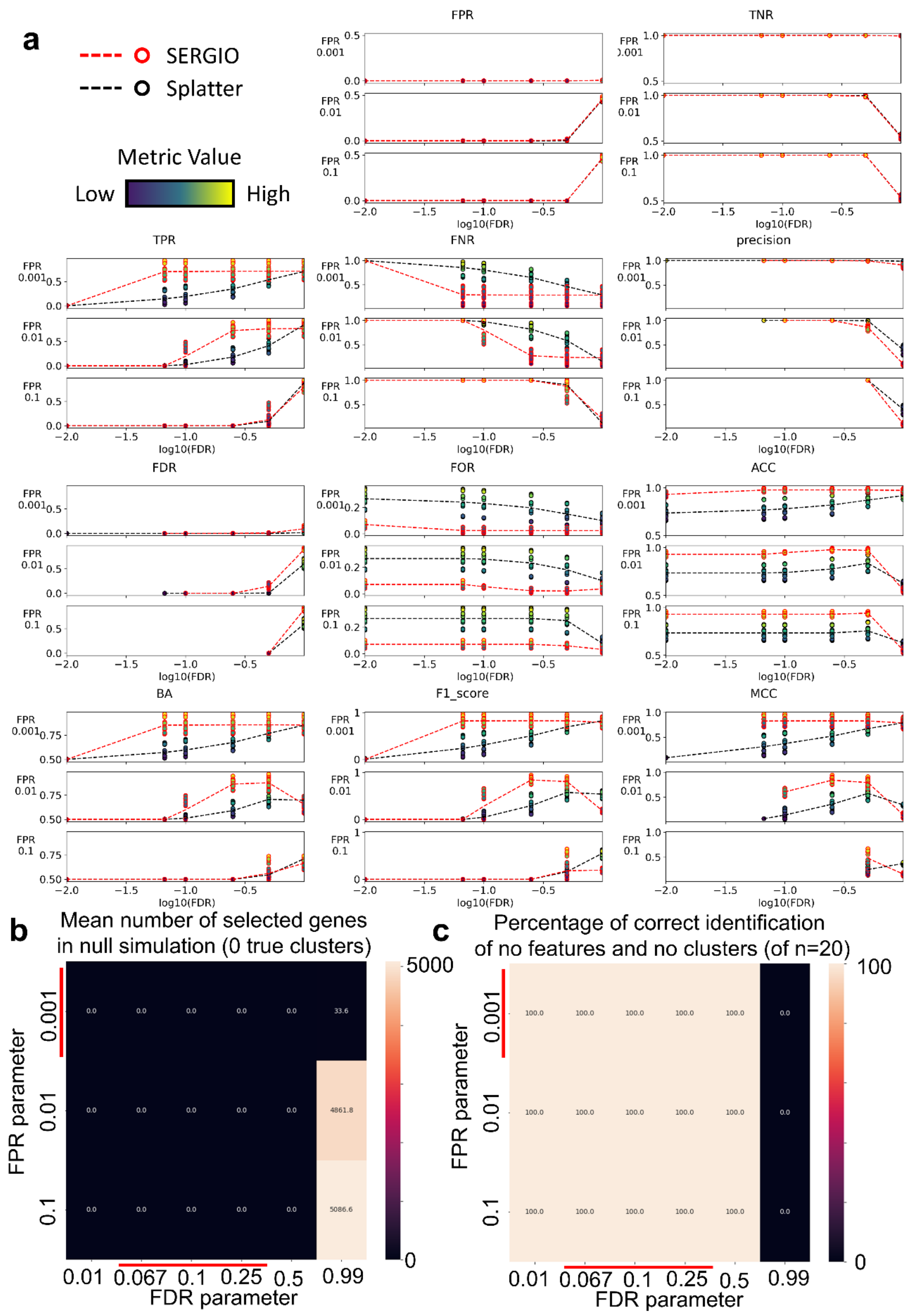

Supplementary Figure 3: Hyperparameter selection with anti-correlation-based feature selection.

a, Scatter plots of 11 different machine learning performance metrics are shown for a hyperparameter sweep of the FDR and FPR settings when running the anti-correlation algorithm. Three FPR settings were used, and are broken into three vertical panels per metric employed (FPR=0.001 (default setting), 0.01, and 0.1). The x-axis of all plots shows the other parameter (FDR), for which 6 values were used (0.01, 0.1, 0.066 (default), 0.25, 0.5, and 0.99). Plots are color-coded by the simulation mode (SERGIO and Splatter). The means of each

metric are plotted across the FDR sweep in lines, stratified by simulation mode (color). **b**, Heatmap showing the average number of selected genes for each combination of the FPR and FDR hyperparameters, n=20 iterations. Red lines indicate recommended hyperparameter ranges. **c**, Heatmap showing the percentage of iterations for which the number of selected genes was 0, therefore indicating a ‘passage’ of the null test; red lines indicate recommended hyperparameter ranges. Source data are provided as a Source Data file.

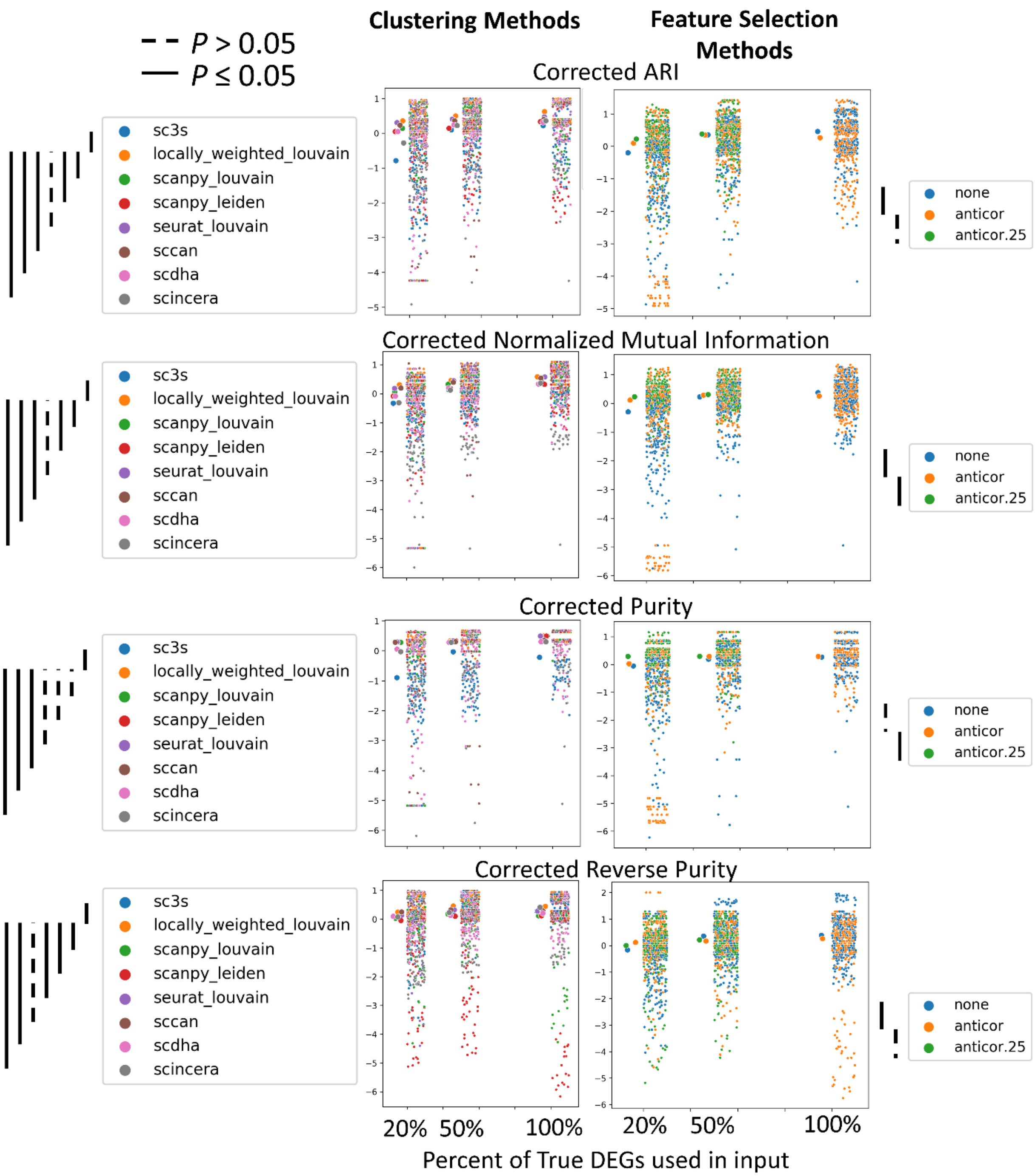

Supplementary Figure 4: Clustering algorithm performance with and without anticorrelation-based feature selection.

Scatter plots showing the relationship between signal to noise in the input (x-axes) and how clustering algorithm and feature selection algorithms impact clustering performance as measured by four metrics (y-axes). In all cases, y-axes show the residuals of a linear model fit after regressing out all other variables, not of interest including number of clusters simulated, simulation program. Y-axes indicate the values of metrics noted in the panel titles, after correction for co-variates. Using Splatter and SERGIO simulations, we benchmarked cluster algorithm performance with no feature selection, feature selection with the default FDR=0.001 hyperparameter, and feature selection with a more sensitive hyperparameter setting (FDR=0.25). Simulations were performed with 3 different values of signal to noise: 1) all (100%) true differentially expressed genes (DEGs) between clusters were included in the input, 2) 50% included, and 3) 20% included, while retaining all randomly expressed genes in all cases, thus varying the input dataset's signal-to-noise ratio.

Displayed points are either all individual measures across all simulations (small spots to the right), or the median (large dots to the left). All non-displayed factors were regressed out for display purposes, but statistics were calculated on a joint model using a mixed-effects linear model (GLM, statistics show deviation from reference group based on GLM fit, statistics are 2-sided). (n=2560 simulations in total across all conditions). Source data are provided as a Source Data file.

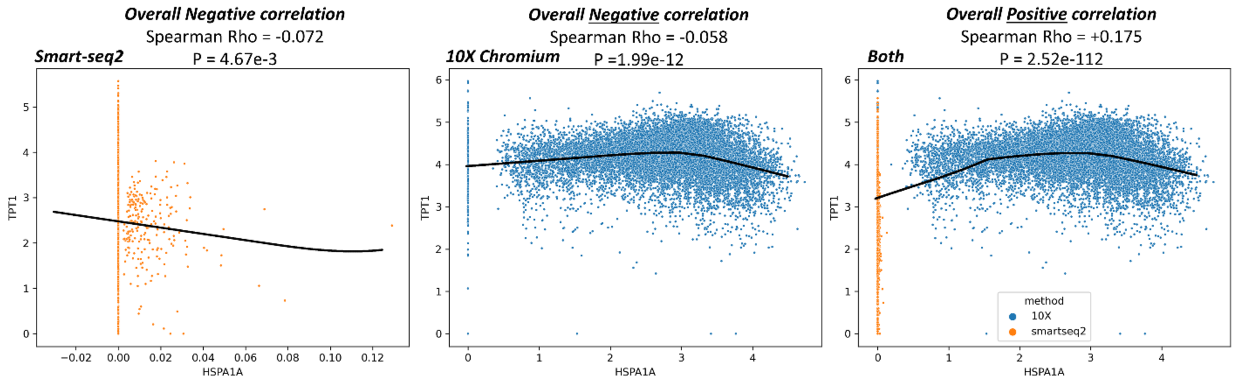

Supplementary Figure 5: Simpson's Paradox is introduced with multi-technology analyses.

An example scatter plot of two genes (*TPT1* and *HSPA1A*) from technical replicates from the Tabula Sapiens processed in parallel using Smart-seq2 and 10X Genomics' Chromium chemistry shows global negative correlations when the two patterns are assessed within a technology. However, when combined, a much more significant positive correlation emerges, strictly from compounding these technical effects over two negative correlations. Spearman rho correlations with p-values are reported. Source data are provided as a Source Data file.

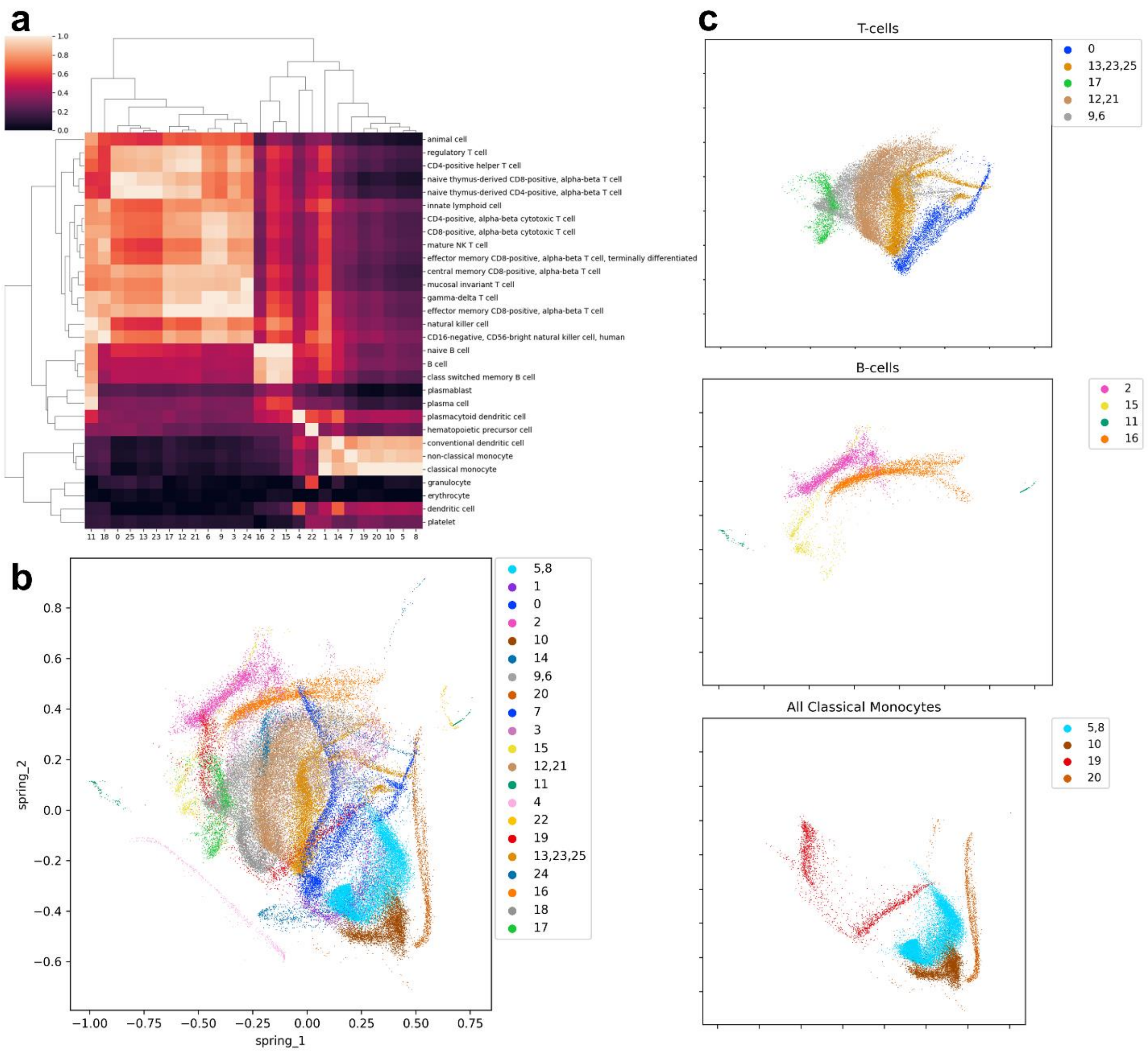

Supplementary Figure 6: First draft cluster labels.

a, A heatmap showing normalized Spearman correlations of the average transcriptomes of clusters discovered here, and in a large reference COVD19 reference dataset. **b**, Spring embedding sowing all first-round clusters. **c**, Spring embedding of several lineages of interest and their corresponding clusters.

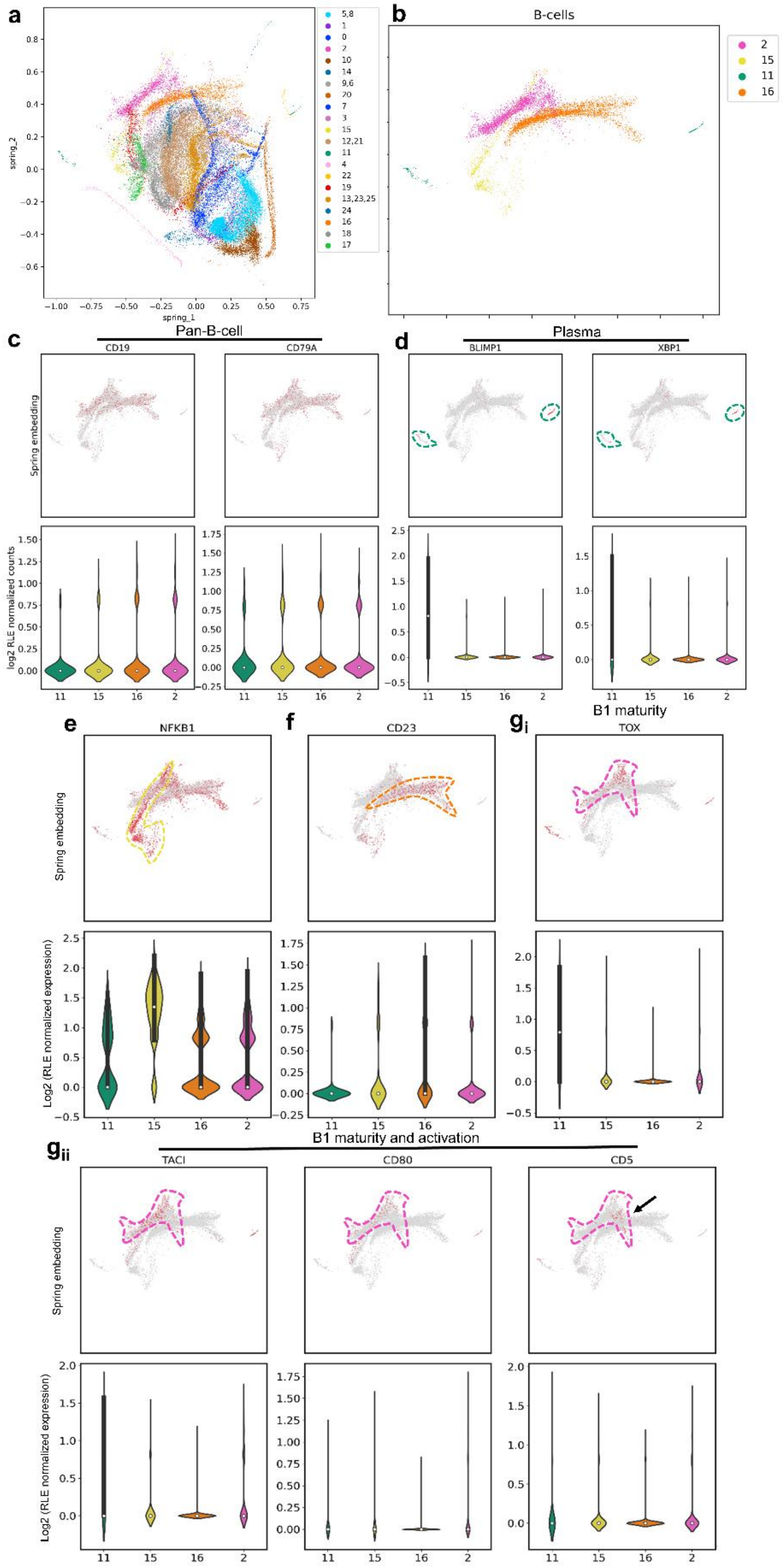

Supplementary Figure 7: Detailed analysis of B-cells.

a, Spring embedding of all clusters. **b,** Spring embedding of B-cell clusters. **c**, Expression of canonical B-cell markers overlayed on the spring embeddings, (higher is red, lower expression is grey), and as violin plots. **d**,

Expression of plasma cell markers *BLIMP1* and *XBP1*. **e**, Expression of *NFKB1*, particularly high in the noted cluster-15, as shown in the spring embedding and violin plots. **f**, *CD23* expression, particularly high in the noted cluster-16, as shown in the spring embedding and violin plots. **g**, B1-like positivity for *TOX*, *TACI*, *CD80*, and *CD5* as shown by expression on spring embeddings and violin plots. Note a small sub-population of *CD5*+ and *CD5*- cells within this cluster. (See **Supplementary Dataset 4** for n per cluster). Boxplots within violin plots show lines that extend to minimum and maximum, with the box bounds from 25th-75th percentile, and center denoting the median.

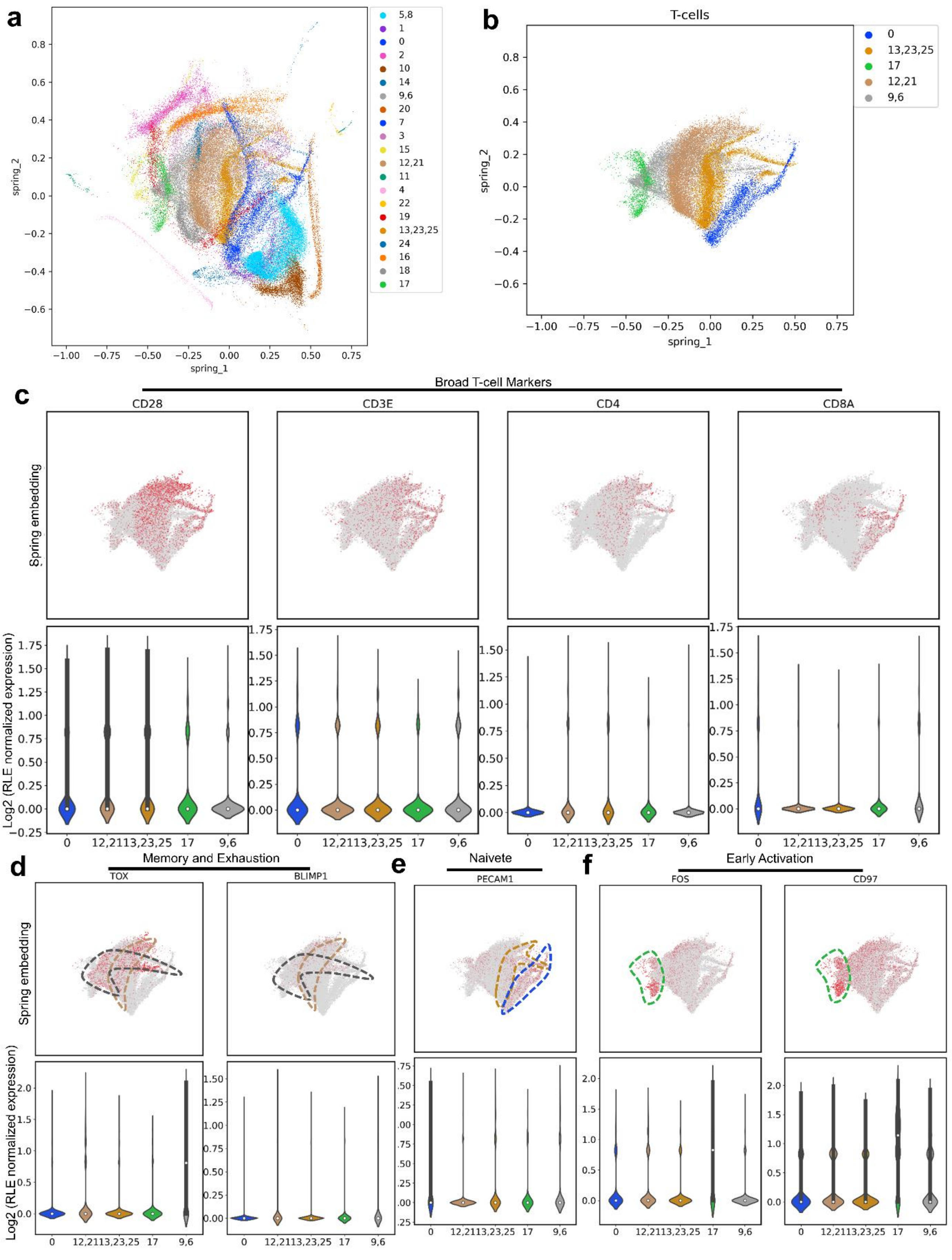

Supplementary Figure 8: Detailed analysis of T-cells.

a, Spring embedding of all clusters. **b**, Spring embedding of T-cell subsets. **c**, Spring embeddings colorized by expression of broad canonical T-cell markers. **d**, Expression of *TOX* and *BLIMP1* indicate memory populations.

e, PECAM1 expression marks naïve populations. **f**, Expression of *FOS* and *CD97* that mark early activation. (See **Supplementary Dataset 4** for n per cluster). Boxplots within violin plots show lines that extend to minimum and maximum, with the box bounds from 25th-75th percentile, and center denoting the median.

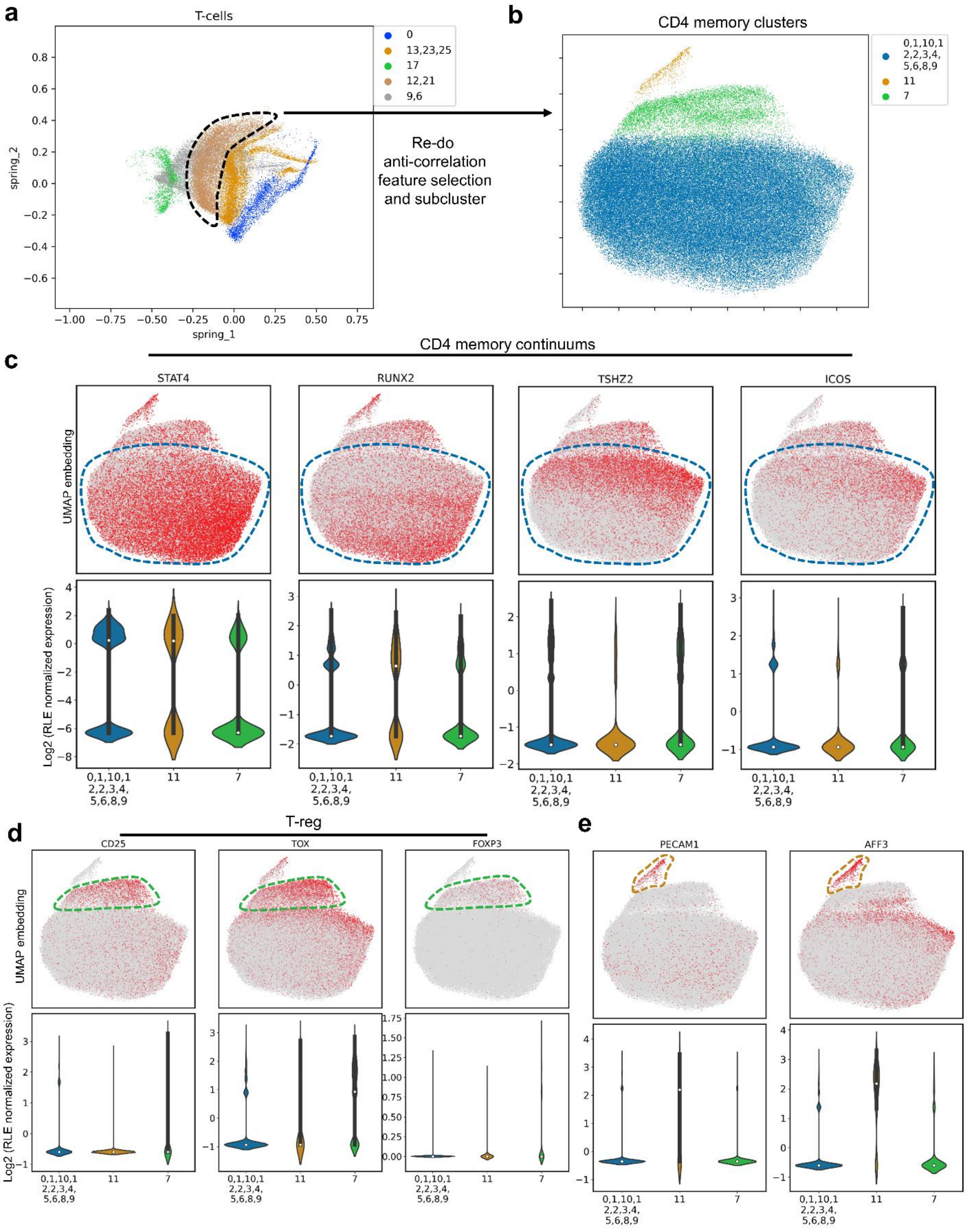

Supplementary Figure 9: Detailed analysis of CD4-memory subsets.

a, Spring embedding of all clusters. **b**, A UMAP projection of the mixed memory CD4 cluster after re-performing feature selection (note that the originally performed spring embedding appeared “knotted” and difficult to see markers of interest due to overlapping populations). **c**, The largest population appeared

activated as shown by the plotted known activation and co-stimulatory markers. **d**, *CD4*+/*CD25*+/*FOXP3*+ T-regs also showed *TOX* positivity. **e**, UMAP showing expression of *PECAM1*+ indicates that the final sub-cluster-11 was likely contaminant naïve cells. (See **Supplementary Dataset 4** for n per cluster). Boxplots within violin plots show lines that extend to minimum and maximum, with the box bounds from 25th-75th percentile, and center denoting the median.

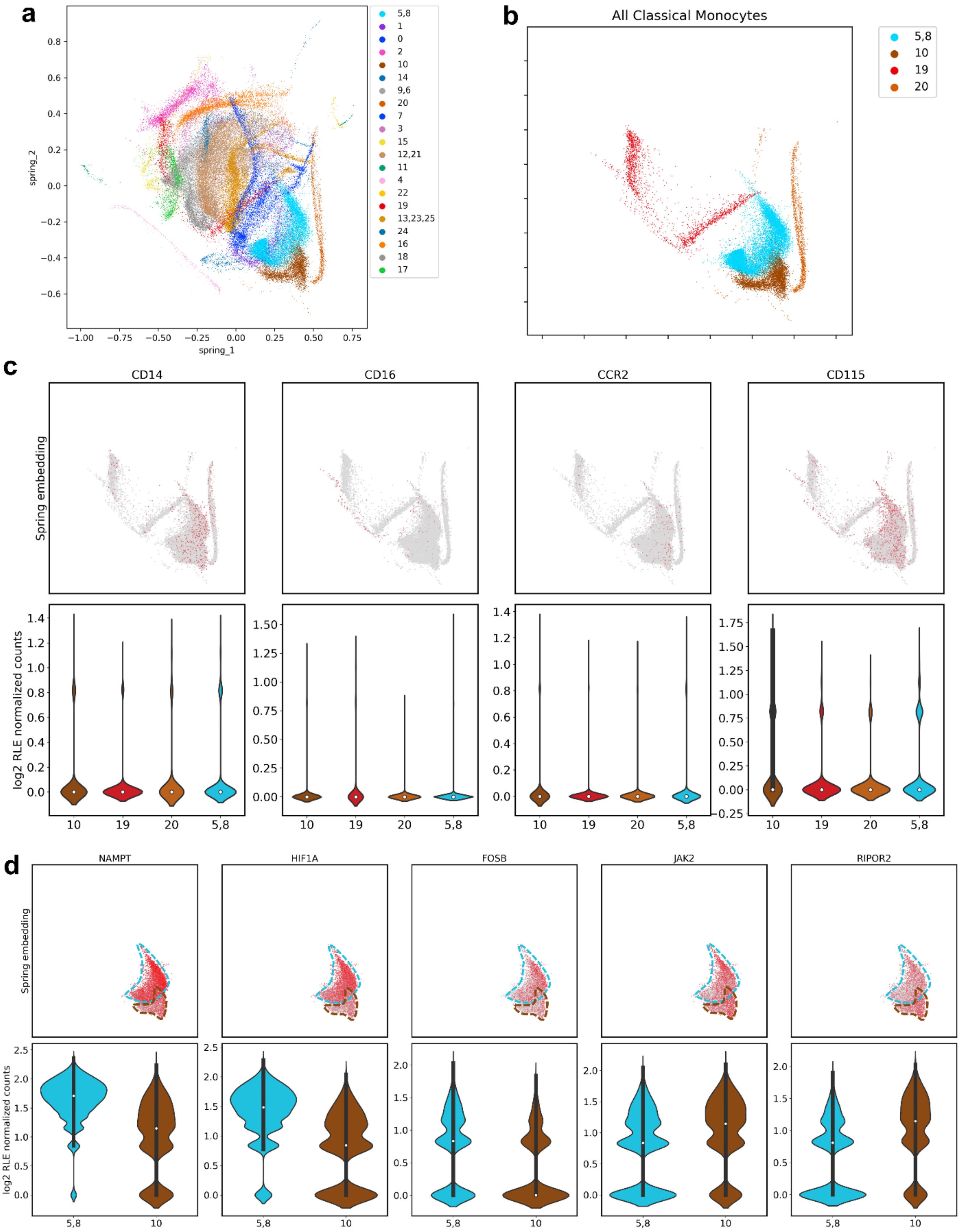

Supplementary Figure 10: Detailed analysis of differentially abundant classical monocyte population.

a, Spring embedding of all clusters. **b**, All clusters labeled as classical monocytes. **c**, Expression of canonical markers for monocytes such as *CD14*, *CD16*, *CCR2*, and *CD115* as shown with colorized spring embeddings

and violin plots. **d**, Expression of several of the top differentially expressed genes between the differentially more abundant cluster-5,8 and the non-differentially-abundant cluster-10, as shown on colorized spring embeddings and violin plots. (See **Supplementary Dataset 4** for n per cluster). Boxplots within violin plots show lines that extend to minimum and maximum, with the box bounds from 25th-75th percentile, and center denoting the median.
