# Supplementary material for: Anti-correlated feature selection prevents false discovery of subpopulations in scRNAseq
Source: Nat Commun. 2024 Jan 24;15:699. doi: 10.1038/s41467-023-43406-9 (PMC10808220; doi:10.1038/s41467-023-43406-9)
Supplement: Supplementary file 3 — Description of Additional Supplementary Files [file 41467_2023_43406_MOESM3_ESM.pdf]

Description of Additional Supplementary Files:

Supplementary Data 1: Symmetric pairwise PMD table, comparing all subjects to each other; subjects with IDs beginning with "D" are type-1 diabetes subjects, while "H" samples are healthy controls. Note that a higher PMD indicates greater dissimilarity, where 1.0 approaches the greatest possible difference, and 0 indicates a high degree of similarity. Reverse PMD (rPMD), is simply 1-PMD, to create a metric more similar to a correlation, where 1 is highly concordant, and 0 is divergent. rPMD was used for plotting the heatmap in Figure 3.

Supplementary Data 2: Cluster by subject contingency table, showing how many cells of each cluster-type were found in each subject.

Supplementary Data 3: Cluster residuals Z-scores, indicate the statistical over-/underabundance relative to an expected null distribution for each subject, given its sampling depth (i.e.: number of observed cells).

Supplementary Data 4: The statistics used for performing differential abundance testing on the PMD standardized residuals (2-sided ANOVA).

Supplementary Data 5: Manual notes on significantly different clusters.

Supplementary Data 6: Mean RLE normalized expression for all manually annotated clusters.

Supplementary Data 7: Mean RLE expression for all subclustered memory CD4 clusters.

Supplementary Data 8: Differentially expressed genes, as determined by Wilcoxon test with Benjamini-Hochberg FDR correction comparing two classical monocyte populations: the differentially more abundant in T1D cluster 5,8 and the similar cluster 10 which was not differentially abundant.
